# Systematic Identification and Functional Analysis of Circular RNAs During Rice Black-Streaked Dwarf Virus Infection in the *Laodelphax striatellus* (Fallén) Midgut

**DOI:** 10.3389/fmicb.2020.588009

**Published:** 2020-09-29

**Authors:** Jianhua Zhang, Haitao Wang, Wei Wu, Yan Dong, Man Wang, Dianshan Yi, Yijun Zhou, Qiufang Xu

**Affiliations:** ^1^Institute of Plant Protection, Jiangsu Academy of Agricultural Sciences, Nanjing, China; ^2^Key Laboratory of Food Quality and Safety of Jiangsu Province – State Key Laboratory Breeding Base, Nanjing, China; ^3^Nanjing Plant Protection and Quarantine Station, Nanjing, China

**Keywords:** circular RNA, RNA-Seq, rice black-streaked dwarf virus, *Laodelphax striatellus*, midgut, circRNA2030

## Abstract

Circular RNAs (circRNAs) are endogenous RNAs that have critical regulatory roles in numerous biological processes. However, it remains largely unknown whether circRNAs are induced in response to plant virus infection in the insect vector of the virus as well as whether the circRNAs regulate virus infection. Rice black-streaked dwarf virus (RBSDV) is transmitted by *Laodelphax striatellus* (Fallén) in a persistent propagative manner and causes severe losses in East Asian countries. To explore the expression and function of circRNAs in the regulation of virus infection, we determined the circRNA expression profile in RBSDV-free or RBSDV-infected *L. striatellus* midgut tissues by RNA-Seq. A total of 2,523 circRNAs were identified, of which thirteen circRNAs were differentially expressed after RBSDV infection. The functions of these differentially circRNAs were predicted by GO and KEGG pathway analyses. The expression changes of five differentially expressed circRNAs and eight parental genes were validated by RT-qPCR. The circRNAs-microRNAs (miRNAs) interaction networks were analyzed and two miRNAs, which were predicted to bind circRNAs, were differentially expressed after virus infection. CircRNA2030 was up-regulated after RBSDV infection in *L. striatellus* midgut. Knockdown of circRNA2030 by RNA interference inhibited the expression of its predicted parental gene phospholipid-transporting ATPase (PTA) and enhanced RBSDV infection in *L. striatellus*. However, none of the six miRNAs predicting to bind circRNA2030 was up-regulated after circRNA2030 knockdown. The results suggested that circRNA2030 might affect RBSDV infection via regulating PTA. Our results reveal the expression profile of circRNAs in *L. striatellus* midgut and provide new insight into the roles of circRNAs in virus–insect vector interaction.

## Introduction

A high-proportion of genomes can be transcribed into RNA and the majority of these RNAs are non-coding RNAs (ncRNAs). The ncRNAs, can be divided into multiple types, including microRNAs (miRNAs), PIWI-interacting RNAs (piRNAs), long non-coding RNAs (lncRNAs), and circular RNAs (circRNAs) (Huttenhofer et al., 2005; Lauressergues et al., 2015). CircRNAs have a single-strand and covalently closed structure. In general, circRNAs are generated through back-splicing the exons and introns of precursor mRNAs and joining a downstream splice donor site with an upstream splice acceptor site (Lasda and Parker, 2014). CircRNAs exist in almost all eukaryotes with abundant and tissue-specific expression patterns (Lasda and Parker, 2014; Salzman, 2016).

An increasing number of researches have shown that circRNAs play vital regulatory roles in numerous biological processes (Gan et al., 2017; Wei et al., 2017). CircRNAs can function as miRNA sponges, splicing interferences, and transcription regulators (Li et al., 2019). For example, circRNA *CDR1as* can impact the development of zebrafish midbrain through sponging miR-7 (Memczak et al., 2013). CircRNAs also play vital roles in host–virus interactions. In *Bombyx mori*, a large number of differentially expressed circRNAs were identified during cytoplasmic polyhedrosis virus (CPV) infection (Hu et al., 2018). The relative expressions of circRNAs and their parent genes were significantly alerted in the IPEC-J2 cell line after porcine endemic diarrhea virus (PEDV) infection (Chen et al., 2019). CircRNAs were involved in the response to cucumber green mottle mosaic virus (CGMMV) infection in watermelon (Sun et al., 2020). In tomato, circRNAs were identified as negative regulators following tomato yellow leaf curl virus (TYLCV) infection (Wang et al., 2018). However, the functions of circRNAs in host–virus interactions are still unclear.

Over 76% of plant viruses are transmitted via insect vectors, including planthoppers, whiteflies, thrips, and aphids (Hogenhout et al., 2008; Dader et al., 2017; Jia et al., 2018). Rice black-streaked dwarf virus (RBSDV) is a member of the genus *Fijivirus* within the family *Reoviridae* and is the causal agent of rice black-streaked dwarf and maize rough dwarf disease (Zhang et al., 2001b; Xu et al., 2014; Wu et al., 2020). RBSDV was first reported in Japan (Kuribayashi and Shinkai, 1952), with the first report in China in 1963 in Yuyao county, Zhejiang Province, and caused severe damage (Chen, 1964; Ren et al., 2016; Liu et al., 2020). RBSDV is transmitted in a persistent propagative manner by *Laodelphax striatellus* (Fallén) (Wu et al., 2020). The genome of RBSDV contains 10 double-stranded RNA (dsRNA) segments (*S1* to *S10*) and encodes 13 proteins (Milne et al., 1973; Zhang et al., 2001a; He et al., 2020). Most RBSDV genomic segments encode one protein, while S5, S7, and S9 encode two proteins. P5-1, P6, and P9-1 proteins encoded by S5, S6, and S9 are the components of the viroplasm (Li et al., 2013; Sun et al., 2013; He et al., 2020). The P10 protein, encoded by S10, is the virus outer capsid protein. P10 can promote RBSDV infection when expressed in rice and can impair the innate immunity in *L. striatellus* (Lu et al., 2019; Zhang et al., 2019).

In its insect vector, RBSDV can move from midgut lumen to hemolymph or other tissues, then to salivary gland, and finally, infect the plant during insect feeding. The midgut is an important barrier for RBSDV infection (Jia et al., 2014). In this research, we investigated the expression profiles of circRNAs in the *L. striatellus* midgut during RBSDV infection by RNA-Seq. The expression of differentially expressed circRNAs and their parental genes was verified by quantitative real-time PCR (RT-qPCR). The functions of the differentially expressed circRNAs were predicted using GO and KEGG analyses. Also, the interactive networks between differentially expressed circRNAs and miRNAs were constructed. Furthermore, the function of circRNA2030 was investigated by RNA interference (RNAi). The results provide new insight into the functions of circRNAs in regulating plant virus infection in the insect vector.

## Materials and Methods

### Insect, Virus, and Sample Preparation

The non-viruliferous populations of *L. striatellus* used in this research were collected from Haian (32.57°N, 120.45°E; Jiangsu Province, China) and maintained in the incubator at 26 ± 1°C with 70%–80% humidity and 16 h light: 8 h dark photoperiod. The RBSDV-infected rice plants with typical dwarf symptom were collected for *L. striatellus* to acquire virus.

The non-viruliferous 3rd-instar nymphs of *L. striatellus* were reared on RBSDV-infected rice plants for 2 days and then transferred to healthy rice seedlings for another 2 days. Finally, the surviving nymphs of *L. striatellus* were collected as RBSDV-infected *L. striatellus*. The non-viruliferous 3rd-instar nymphs of *L. striatellus* were reared on healthy rice seedlings for 4 days, and the surviving nymphs of *L. striatellus* were collected as virus-free *L. striatellus*.

RBSDV-free (VF) and RBSDV-infected (RB) midguts were collected from 200 non-viruliferous or RBSDV-infected *L. striatellus* nymphs for RNA-Seq analysis. For midgut dissection, the nymphs were rinsed with 75% ethanol and then washed three times using sterilized-deionized water. The midgut tissue was dissected in chilled 1× phosphate-buffered saline (1× PBS, pH 7.4) with sterile forceps under a stereomicroscope. Each sample contained three independent biological repetitions.

### RNA Extraction and RNA-Seq

Total RNA of *L. striatellus* midgut was extracted using TRIzol reagent (Invitrogen, United States) according to the manufacturer’s instructions. The quantity and quality of total RNA was determined by spectrophotometry (NanoDrop 2000, Thermo Scientific) and agarose gel electrophoresis, respectively.

Ribosomal RNA in the total RNAs was eliminated by Epicentre Ribo-Zero Gold Kit (Illumina, United States). Thereafter, the cDNA libraries were constructed by the mRNA-Seq sample preparation kit using approximately 10 μg of RNA (Illumina, United States). Sequencing was carried out using the Illumina HiSeq 4000 platform, and the whole transcriptome was performed by Lianchuan Biotechnology (Hangzhou, China).

### Bioinformatics Analysis

The sequence quality was analyzed by FastQC after removal of the reads containing low-quality bases, undetermined bases, and adaptor contamination^1^. Bowtie2, Tophat2, and tophat-fusion were used to map sequences to the genome of *L. striatellus* (GenBank No.: GCA_003335185.2) (Kim and Salzberg, 2011; Langmead and Salzberg, 2012; Kim et al., 2013). The mapped reads were de novo assembled to circRNAs using CIRCExplorer (Zhang et al., 2014, 2016; Gao et al., 2015). The unmapped reads were used to identify back splicing reads by CIRCExplorer and tophat-fusion (Kim and Salzberg, 2011; Zhang et al., 2014, 2016; Gao et al., 2015). The expression levels of circRNAs were normalized using the formula (normalized expression = (mapped reads)/(total reads) × 1,000,000). Only the comparisons with *p* value < 0.05 were regarded as having significant differential expression by the R package (Robinson et al., 2010). Gene Ontology (GO^2^) and Kyoto Encyclopedia of Genes and Genomes database (KEGG^3^) were performed to analyze the potential functions of circRNAs (Kanehisa et al., 2008; Young et al., 2010). The miRanda software^4^ was used to detect the potential binding miRNAs of these differentially expressed circRNAs.

### RT-qPCR

The cDNA was synthesized from 1 μg of total RNA with PrimeScript^TM^ RT reagent kit with gDNA Eraser (Takara, Japan). SYBR PrimeScript^TM^ RT-PCR Kit (Takara, Japan) and IQ^TM^5 multicolor real-time PCR detection system (BIO-RAD, United States) were used and each experiment included three independent technical and biological replications. RT-qPCR was carried out for 40 cycles (94°C for 5 s, 60°C for 34 s) after an initial denaturation step (94°C for 30 s). RPL5 (encoding ribosome protein L5) was used as an internal reference gene (Wu et al., 2019a). U6 snRNA was used as an internal reference for miRNAs analysis (Wu et al., 2019b). The data were calculated by 2^–ΔΔ*Ct*^ method. The primers were designed with Beacon Designer 7.7 and listed in Supplementary Table S1.

### RNA Interference

The T7 high yield transcription kit (Invitrogen, America) was used to synthesize double-stranded RNA (dsRNA). The RBSDV-free 3rd-instar nymphs of *L. striatellus* were immobilized on a 1% agarose plate and approximately 100 nL dsRNA (2 μg/μL) was injected into the conjunction between the prothorax and mesothorax via capillary on FemtoJet (Eppendorf, Germany) after being anesthetized with carbon dioxide. The dsRNA of enhanced green fluorescent protein (EGFP) was injected as a control. Each experiment was carried out with 30 nymphs and three independent biological repetitions.

The RNAi efficiency of circRNA2030 was determined by calculating its relative expression at 1, 3, and 5 days after injection of circRNA2030 dsRNA. Two days after microinjection, the nymphs of *L. striatellus* were reared on RBSDV-infected rice plants for 2 days. Then, the nymphs were transferred to healthy rice seedlings for another 2 days, and the RBSDV accumulation was determined by detecting the gene and protein expression of P10 using RT-qPCR and immunofluorescence, respectively.

### Immunostaining

The midgut tissue was dissected in chilled 1× PBS under a stereomicroscope and fixed with 4% paraformaldehyde for 1 h. Then, the midguts were permeabilized with 2% Triton X-100 for 30 min and blocked with 3% BSA for 2 h. The primary antibody (anti-RBSDV P10 mouse mAb conjugated with FITC) was incubated with the midguts at 4°C overnight. Then, the midguts were incubated with TRITC phalloidin (Solarbio, China) for 10 min. An LSM 710 (ZEISS, Germany) was used to view the confocal imaging.

### Statistical Analysis

SPSS 20.0 (IBM Corporation, United States) was used to perform the statistical analysis. One-way analysis of variance (ANOVA) with least significant difference (LSD) test was used in comparing the gene expression levels. The *p*-value < 0.05 and *p*-value < 0.01 were regarded as significant and very significant differences, respectively.

## Results

### Profiling of CircRNAs in *L. striatellus* Midguts in Response to RBSDV Infection

Six libraries were constructed from RBSDV-free (VF) and RBSDV-infected (RB) *L. striatellus* midguts and analyzed by RNA-Seq. The sequence was deposited in the National Center for Biotechnology Information (NCBI) with accession number GSE153102. A total of 2,523 circRNAs were identified (Supplementary Table S2). In this study, the length of circRNAs ranged from 116 to 121,200 bp and 86.76% of circRNAs were greater than 1,000 bp (Supplementary Figure S1A). Source statistics showed that about 98% of the circRNAs were exonic circRNAs, and about 2% of the circRNAs were from introns (Supplementary Figure S1B). Due to the limitations of CIRCExplorer software analysis, none circRNAs were identified from intergenic exons and introns.

### Analysis of Differentially Expressed CircRNAs

**FIGURE 1:**
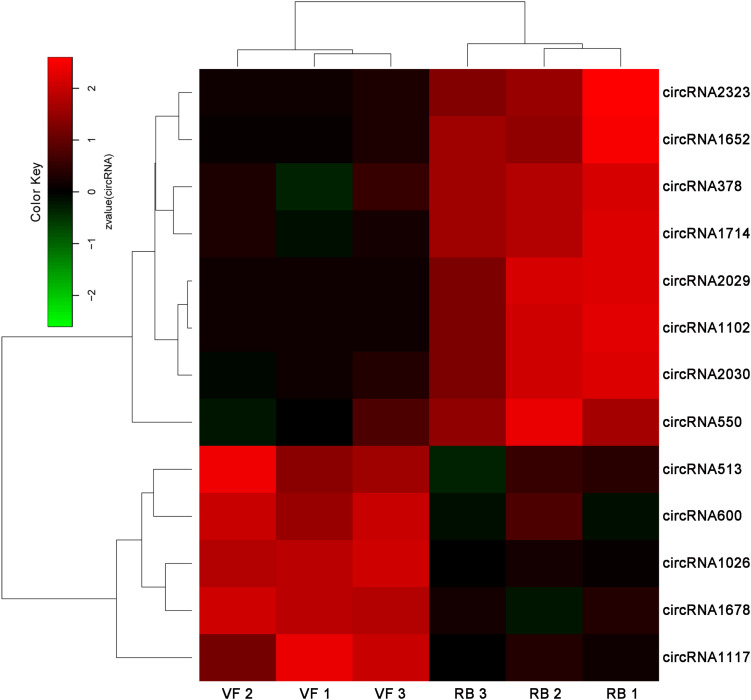
The heatmap of the 13 differentially expressed circRNAs in VF and RB *L. striatellus* midgut. The expression changes of differentially expressed circRNAs are presented by log2^(fold changes)^. VF, virus-free; RB, RBSDV infected.

**FIGURE 2:**
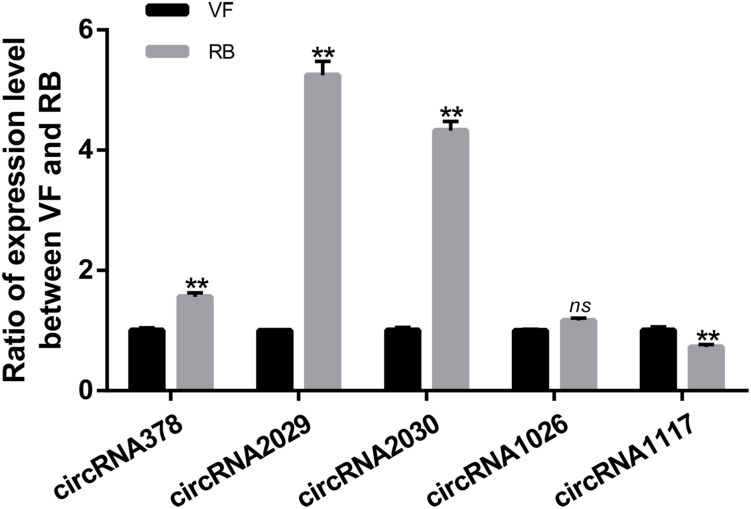
Validation of the expression changes of five differentially expressed circRNAs by RT-qPCR in VF and RB *L. striatellus* midgut. The expressions of five differentially expressed circRNAs were analyzed through 2^–ΔΔ*Ct*^ method. The *ns* represents no significant difference, and the asterisks (**) indicate significant differences at *p* < 0.01 levels.

Thirteen circRNAs were differentially expressed in response to RBSDV infection in *L. striatellus* midguts, including eight up-regulated and five down-regulated circRNAs (Supplementary Table S3). Heat maps were generated to illustrate the expression patterns of the 13 differentially expressed circRNAs (Figure 1). We randomly selected three up-regulated (circRNA378, circRNA2029, and circRNA2030) and two down-regulated circRNAs (circRNA1026 and circRNA1117) to verify the circRNA expression by RT-qPCR. The expression levels of circRNA378, circRNA2029, and circRNA2030 were confirmed to be up-regulated and the expression level of circRNA1117 was down-regulated in *L. striatellus* midgut after RBSDV infection (Figure 2). These data were consistent with the transcriptome. However, the expression level of circRNA1026 did not have any changes after RBSDV infection (Figure 2).

### GO and KEGG Pathway Analysis of Differentially Expressed CircRNAs

**FIGURE 3:**
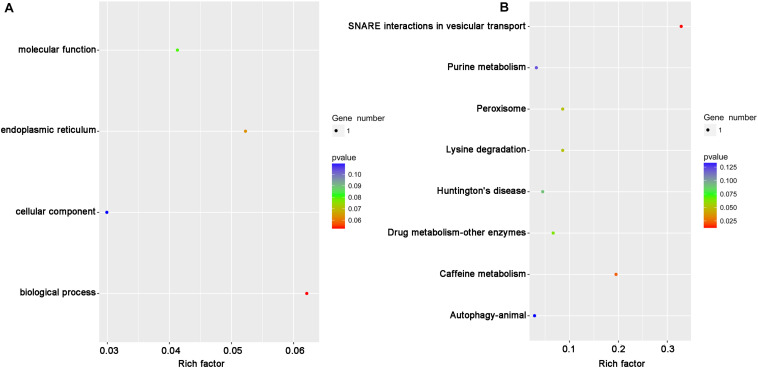
The GO and KEGG pathway analysis of the parental genes of differentially expressed circRNAs. **(A)** The top four GO terms of parental genes were analyzed. **(B)** The top eight enriched pathways for the parental genes were analyzed. GO: gene ontology; KEGG: Kyoto Encyclopedia of Genes and Genomes.

**FIGURE 4:**
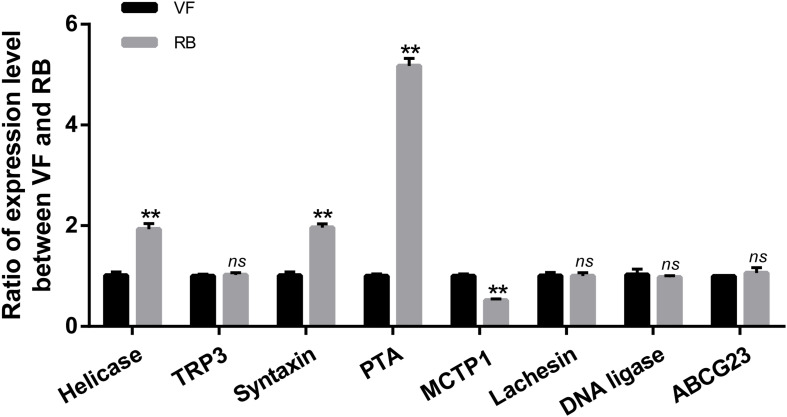
Validation of the expression of eight parental genes by RT-qPCR. The *ns* represents no significant difference, and the asterisks (**) indicate significant differences at *p* < 0.01 levels.

It has been reported that the function of circRNAs can be suggested through characterizing the features of parental linear mRNAs (Wei et al., 2017). In this study, 13 differentially expressed circRNAs were predicted from 11 parental genes (Supplementary Table S4). To predict the functions of these differentially expressed circRNAs, the GO and KEGG analyses of their parental genes were performed. GO analysis revealed that the biological process was the most significantly different subgroup (Figure 3A). SNARE interactions in vesicular transport and caffeine metabolism were shown as the top two most significantly enriched pathways in KEGG analysis (Figure 3B). To clarify whether the parental genes of the differentially expressed circRNAs are also induced in response to RBSDV infection, the expression patterns of eight parental genes were analyzed by RT-qPCR. As shown in Figure 4, the expression levels of four parental genes, helicase, syntaxin, phospholipid-transporting ATPase (PTA) and multiple C2 and transmembrane domain-containing protein 1 (MCTP1) were differentially expressed. In contrast, the expression of other parental genes did not change after RBSDV infection.

### CircRNAs-MiRNAs Interaction Analysis

**FIGURE 5:**
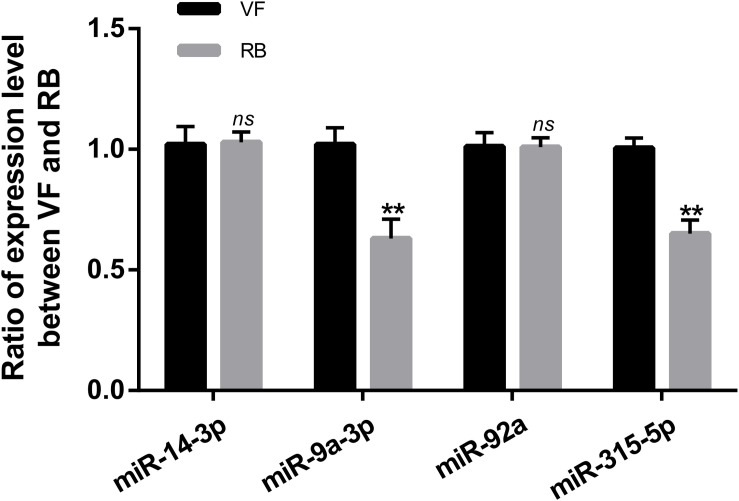
Validation of the expression of four microRNAs by RT-qPCR. The *ns* represents no significant difference, and the asterisks (**) indicate significant differences at *p* < 0.01 levels.

CircRNAs can act as miRNA sponges to regulate the post-transcriptional level of miRNA’s target genes (Li et al., 2019). In this study, the interactions of differentially expressed circRNAs-miRNAs were analyzed according to the miRanda software. Thirteen differentially expressed circRNAs were predicted to bind 30 miRNAs (Table 1). Both circRNA1102 and circRNA2030 were predicted to bind six miRNAs. Four miRNAs, miR-14-3p, miR-9a-3p, miR-92a, and miR-315-5p, were predicted to bind two or more differentially expressed circRNAs. Besides, miR-9a-3p and miR-315-5p were significantly down-regulated, and the expression levels of miR-14-3p and miR-92a did not change after RBSDV infection (Figure 5).

**TABLE 1:** The miRNAs predicted to bind the differentially expressed circRNAs.

Accession name	Exons	Putative miRNAs	miRanda score	miRanda energy
circRNA2323	4	miR-6497-3p	148.00	−15.32
circRNA378	2	miR-79	170.00	−13.69
		miR-305-5p	156.00	−15.86
		miR-79-3p	170.00	−13.69
		miR-9a-3p	159.00	−14.68
circRNA1714	2	miR-971	145.00	−12.45
		miR-137-5p	149.00	−14.55
		miR-1-3p	157.00	−17.34
		miR-14-3p	142.00	−11.82
		miR-1	157.00	−17.34
circRNA2029	3	miR-124-3p	154.00	−17.65
		miR-315-5p	147.00	−10.32
		miR-965-3p	141.00	−10.52
circRNA1102	4	miR-6497-5p	142.00	−15.44
		miR-92a	147.00	−12.08
		miR-92a-3p	152.00	−13.79
		miR-9a-3p	150.00	−11.67
		miR-92b-3p	152.00	−13.32
		miR-92-3p	156.00	−19.08
circRNA2030	3	miR-8-3p	146.00	−16.39
		miR-184-3p	153.00	−20.27
		miR-277-3p	163.00	−19.18
		miR-315-5p	147.00	−10.32
		miR-277	163.00	−19.18
		miR-932	148.00	−14.93
circRNA513	6	miR-252a-3p	146.00	−24.25
		miR-993a-3p	140.00	−15.24
		miR-14-3p	147.00	−10.80
		miR-34	156.00	−28.51
circRNA600	2	miR-971	158.00	−15.59
		miR-1000	148.00	−15.02
		miR-14-3p	157.00	−15.26
circRNA1026	5	miR-210	154.00	−25.91
		miR-993a-3p	151.00	−14.91
		miR-210-3p	154.00	−25.91
circRNA1678	2	miR-92a	145.00	−13.81
		miR-14-3p	170.00	−21.30
circRNA1117	2	miR-2765	149.00	−16.96

### CircRNA2030 Regulated RBSDV Infection in *L. striatellus* Midgut

**FIGURE 6:**
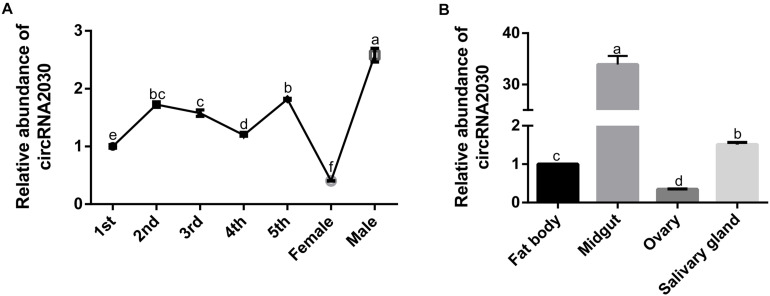
The expressions of circRNA2030 in different developmental stages and different tissues of *L. striatellus*. **(A)** CircRNA2030 was expressed in all developmental stages and had the highest level in the male adult. **(B)** CircRNA2030 was highly expressed in the midgut. The letters a, b, c and d present significant differences (*p* < 0.05) of the expression level of circRNA2030 at different developmental stages and in different tissues.

**FIGURE 7:**
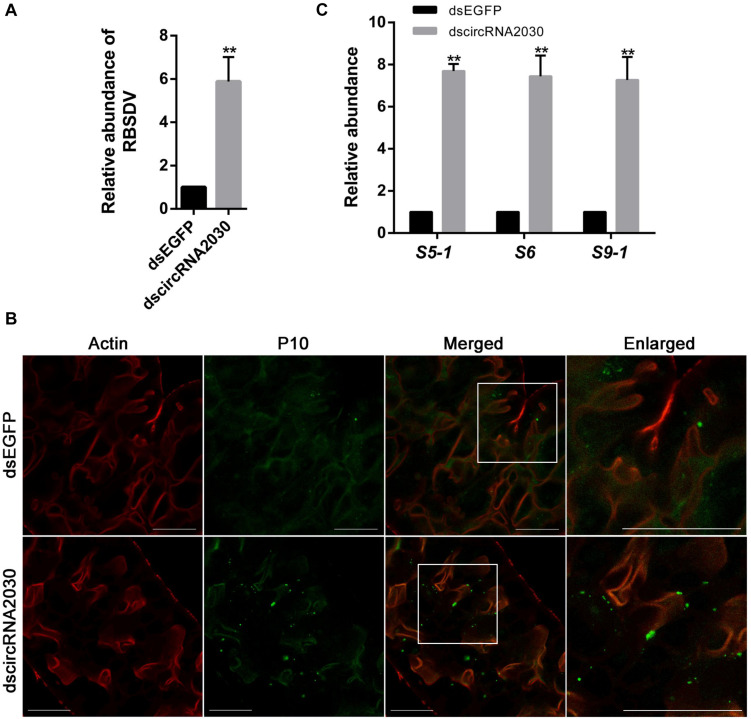
Knockdown of circRNA2030 increased RBSDV accumulation in *L. striatellus* midgut. **(A)** RT-qPCR analyzed the expression of RBSDV *S10* after knockdown of circRNA2030 in *L. striatellus* midgut. The expression level of *S10* was used as an indicator of relative abundance of RBSDV. **(B)** Immunofluorescence analysis showed that the relative abundance of RBSDV, labeling by antibody against P10 protein and presenting as green dots, was significantly increased after knockdown of circRNA2030. **(C)** The relative expression levels of *S5-1*, *S6*, and *S9-1* genes were significantly increased after knockdown of circRNA2030. The asterisks (**) indicate significant differences at *p* < 0.01 levels. Scale bars, 50 μm.

The expression of circRNA2030 changed more than 4.0-fold after RBSDV infection. It was predicted to bind six miRNAs. We further studied its functions in RBSDV infection of *L. striatellus* midgut. The expression patterns of circRNA2030 were examined in different developmental stages, from 1st-instar to adult and in different tissues, including fat body, midgut, ovary, and salivary gland. The expression of circRNA2030 was observed in all developmental stages and had highest level in the male adult (Figure 6A). The tissue expression patterns showed that circRNA2030 was highly expressed in the midgut (Figure 6B). The relative expression level of circRNA2030 was significantly decreased three and five days after dsRNA injection (Supplementary Figure S2). Knockdown of circRNA2030 significantly increased the expression of RBSDV *S10* in *L. striatellus* midgut, suggesting that inhibition of circRNA2030 promotes RBSDV accumulation (Figure 7A). Besides, immunofluorescence analysis also showed that RBSDV accumulation in *L. striatellus* midgut was also increased after knockdown of circRNA2030 (Figure 7B). Furthermore, the relative expression levels of RBSDV replication related genes, *S5-1*, *S6*, and *S9-1*, were significantly up-regulated after knockdown of circRNA2030 (Figure 7C).

**FIGURE 8:**
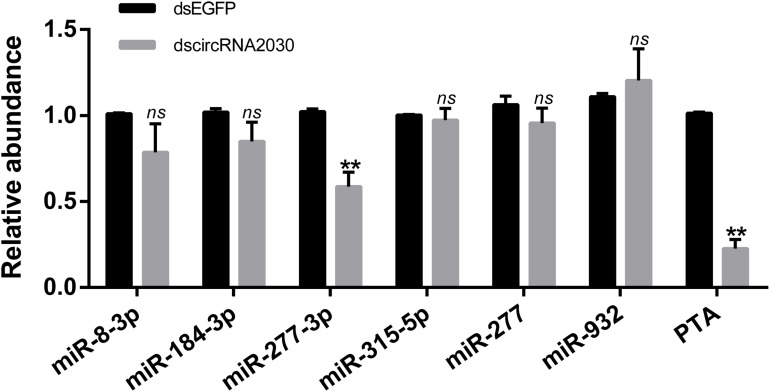
Validation the relationships among the circRNA2030 and its related miRNAs (miR-8-3p, miR-184-3p, miR-277-3p, miR-315-5p, miR-277, and miR-932) and parental gene (PTA) by RT-qPCR. The *ns* represents no significant difference, and the asterisks (**) indicate significant differences at *p* < 0.01 levels.

In order to explore whether circRNA2030 regulates RBSDV infection via miRNA or its parental gene, the relationships between circRNA2030 and its related miRNAs (miR-8-3p, miR-184-3p, miR-277-3p, miR-315-5p, miR-277, and miR-932) or its parental gene (PTA) were investigated. In general, if circRNA2030 could act as miRNAs sponges, the expression levels of the adsorbed miRNAs should be up-regulated when circRNA2030 was knocked down. However, none of the six related miRNAs was up-regulated after knockdown of circRNA2030 (Figure 8). In the other hand, the expression of PTA was significantly down-regulated after knockdown of circRNA2030 (Figure 8). In addition, the relative expression level of PTA was significantly up-regulated after RBSDV infection (Figure 4). These results indicated that circRNA2030 may regulate RBSDV infection via regulating the expression of PTA at the mRNA level.

## Discussion

With the novel computational methodology and the advent of deep sequencing technology, numerous circRNAs have been identified in eukaryotes, including mice, *Caenorhabditis elegans*, and plants (Memczak et al., 2013; Westholm et al., 2014; Ye et al., 2015; Cortes-Lopez et al., 2018). However, functional studies of circRNAs in insects are still at preliminary stages. Recently, significant progress has been made in the identification of circRNAs in insects, especially in *Drosophila melanogaster* and *B. mori* (Westholm et al., 2014; Gan et al., 2017). It has been reported that circRNAs participate in the responses of viral infection in animal and plant, such as CPV infection in *B. mori* (Hu et al., 2018), and TYLCV infection in tomato (Wang et al., 2018). However, few studies focus on the expression profiles and functions of circRNAs in plant virus–insect vector interactions. Midgut is an important barrier for persistent circulative plant viruses to infect insect vectors (Jia et al., 2018; Qin et al., 2018). Our present research characterizes the comprehensive types and expression patterns of circRNAs in *L. striatellus* midgut during RBSDV infection for the first time. After verifying the sequence quality with FastQC, a total of 2,523 circRNAs were systematically identified in virus-free and RBSDV-infected *L. striatellus* midgut. The total number of the circRNAs identified in our research was similar to other insect species, such as *D. melanogaster* and *B. mori* (Westholm et al., 2014; Gan et al., 2017). The length of 86.76% circRNAs was over 1,000 bp in this study. Although, it is usually reported that the length of circRNA is less than 1,000 bp, there are still many circRNAs longer than 1,000 bp, even longer than 10,000 bp in circBase^5^. Also, 13 differentially expressed circRNAs were obtained after RBSDV infection. Mainly, the identification and quantification of circRNAs were dependent on the reverse splicing reads, resulting in a large intra-group difference in the identified circRNAs. Therefore, the number of differentially expressed circRNAs was quite less in this study. Altogether, these data will be helpful to identify specific circRNAs involved in virus infection in the insect vector.

Understanding the functions of parental linear mRNAs will be helpful to reveal the roles of circRNAs (Wei et al., 2017). The differentially expressed circRNAs identified in our study were predicted from 11 parental genes and were mainly predicted to be involved in SNARE interactions in vesicular transport and caffeine metabolism by KEGG analysis and biological process by GO analysis. The expression changes of some circRNAs were verified by RT-qPCR, and most of the results were consistent with the sequencing, indicating the accuracy results of RNA-Seq were reliable. Among the parental genes of the circRNAs, helicase, syntaxin, and PTA were significantly up-regulated, and MCTP1 was significantly down-regulated after RBSDV infection. It has been reported that syntaxin is involved in the release of hepatitis C virus (HCV) through mediating vesicles fusion (Ren et al., 2017). These differentially expressed parental genes might be involved in the infection of RBSDV in *L. striatellus* midgut. However, a relationship between circRNAs forms and their parental genes needs to be studied.

A growing body of research has shown that circRNAs function as miRNA sponges to regulate the post-transcriptional level of gene expression (Li et al., 2019). To further explore the roles of the differentially expressed circRNAs during RBSDV infection, a total of 30 miRNAs were predicted to bind to the circRNAs. A single miRNA was predicted to combine with several circRNAs. For example, miR-14-3p could bind by four differentially expressed circRNAs. It has been reported that miR-14 can regulate various ecdysone-signaling pathway genes to switch off ecdysone production after ecdysis in silkworm (He et al., 2019). However, the expression level of miR-14-3p (measured by RT-qPCR) was not changed after RBSDV infection, indicating that miR-14-3p may not be directly involved in virus infection, but it might affect the development of the insect vector. In addition, we found that the expression levels of miR-9a-3p and miR-315-5p were significantly down-regulated after RBSDV infection. miR-9a is involved in the dengue virus (DENV) infection in mosquitoes (Avila-Bonilla et al., 2017). In shrimp, miR-315 could facilitate the white spot syndrome virus (WSSV) infection by targeting the expression of the gene encoding the prophenoloxidase-activating enzyme to attenuate phenoloxidase activity (Jaree et al., 2018). The results suggest that miR-9a-3p and miR-315-5p may be involved in RBSDV infection, and the mechanism is worth further study. In addition, a network of circRNA-miRNA is presented in *L. striatellus* midgut following RBSDV infection. Altogether, our results provide a new insight into the mechanism of plant virus-insect vector interactions.

CircRNA2030 was significantly expressed after RBSDV infection and bound to six miRNAs. To further study its functions in virus infection, we analyze its expression pattern. It was expressed in all developmental stages and mainly expressed in the midgut of *L. striatellus*, indicating a key biological role of circRNA2030 in the midgut. Knockdown of circRNA2030 caused a significant increase of RBSDV accumulation in *L. striatellus* midgut, indicating that circRNA2030 has antivirus functions in *L. striatellus* under RBSDV infection. To our knowledge, this is the first report that circRNA can regulate plant virus infection in an insect vector. To explore the possible molecular mechanism of circRNA2030 regulating RBSDV infection, the relationships among the circRNA2030 and its related miRNAs and parental gene were investigated. The results indicated that the possible molecular mechanism of circRNA2030 regulating RBSDV infection might be via regulating the expression of PTA at the mRNA level.

## Conclusion

The expression profile and the differentially expressed circRNAs were identified in *L. striatellus* midgut after RBSDV infection, and the function of circRNA2030 in RBSDV infection was analyzed. These results suggest that circRNAs play pivotal roles for virus infection through the insect vector.

## Data Availability Statement

The datasets presented in this study can be found in online repositories. The names of the repository/repositories and accession number(s) can be found in the article/ Supplementary Material.

## Author Contributions

JZ and QX conceived and designed the experiments. JZ, HW, WW, YD, and MW performed the experiments. JZ, HW, DY, and QX analyzed the data. JZ, HW, YZ, and QX wrote the manuscript. All authors contributed to the article and approved the submitted version.

## Conflict of Interest

The authors declare that the research was conducted in the absence of any commercial or financial relationships that could be construed as a potential conflict of interest.

## Footnotes

1 http://www.bioinformatics.babraham.ac.uk/projects/fastqc/

2 http://www.geneontology.org/

3 http://www.kegg.jp

4 http://www.microrna.org/microrna/home.do

5 http://www.circbase.org/

## Supplementary Material

Supplementary Figure 1 The length and source statistics of the 2,523 circRNAs identified from VF and RB *L. striatellus* midgut. **(A)** Length distribution of circRNAs. **(B)** Source statistics of circRNAs.

Supplementary Figure 2 Analysis of the expression level of circRNA2030 after dsRNA injection by RT-qPCR. The *ns* represents no significant difference, and the asterisks (^∗∗^) indicate significant differences at *p* < 0.01 levels.
