# Supplementary figures and images for: Systematic Identification and Functional Analysis of Circular RNAs During Rice Black-Streaked Dwarf Virus Infection in the Laodelphax striatellus (Fallén) Midgut
Source: Front Microbiol. 2020 Sep 29;11:588009. doi: 10.3389/fmicb.2020.588009 (PMC7550742; doi:10.3389/fmicb.2020.588009)

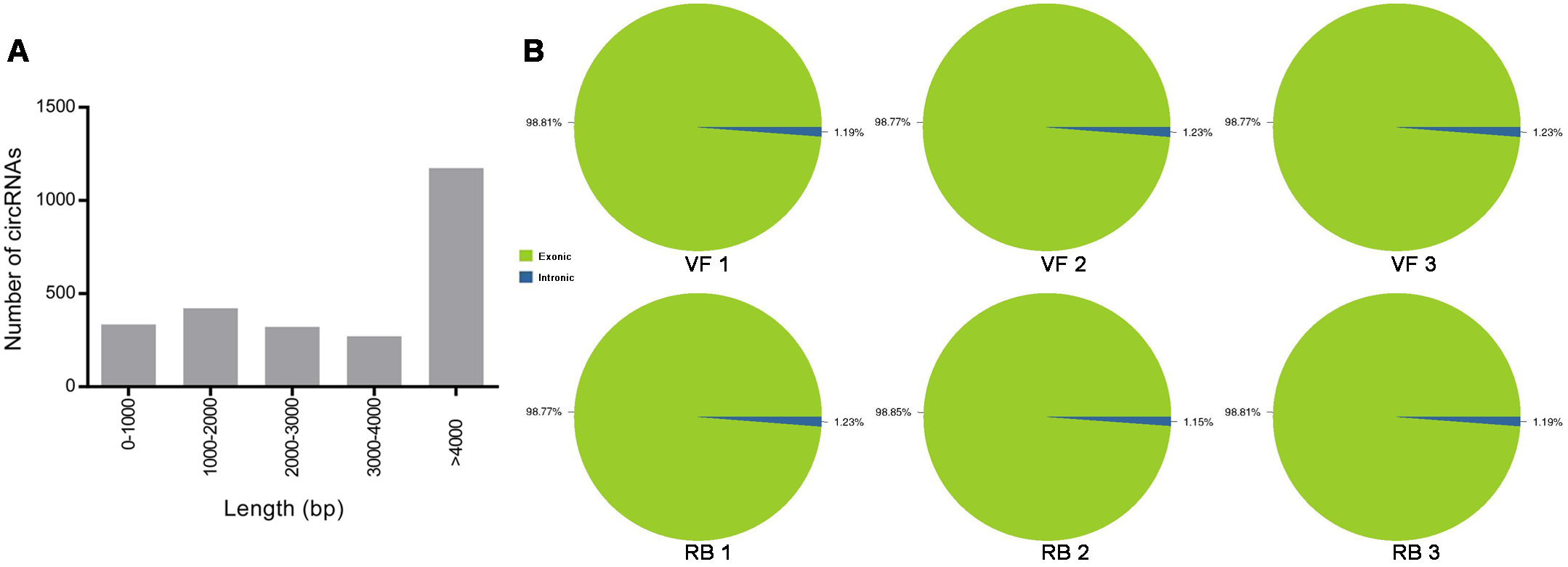

Supplement: Supplementary Figure 1 — The length and source statistics of the 2,523 circRNAs identified from VF and RB L. striatellus midgut. (A) Length distribution of circRNAs. (B) Source statistics of circRNAs. [file Image_1.TIF]

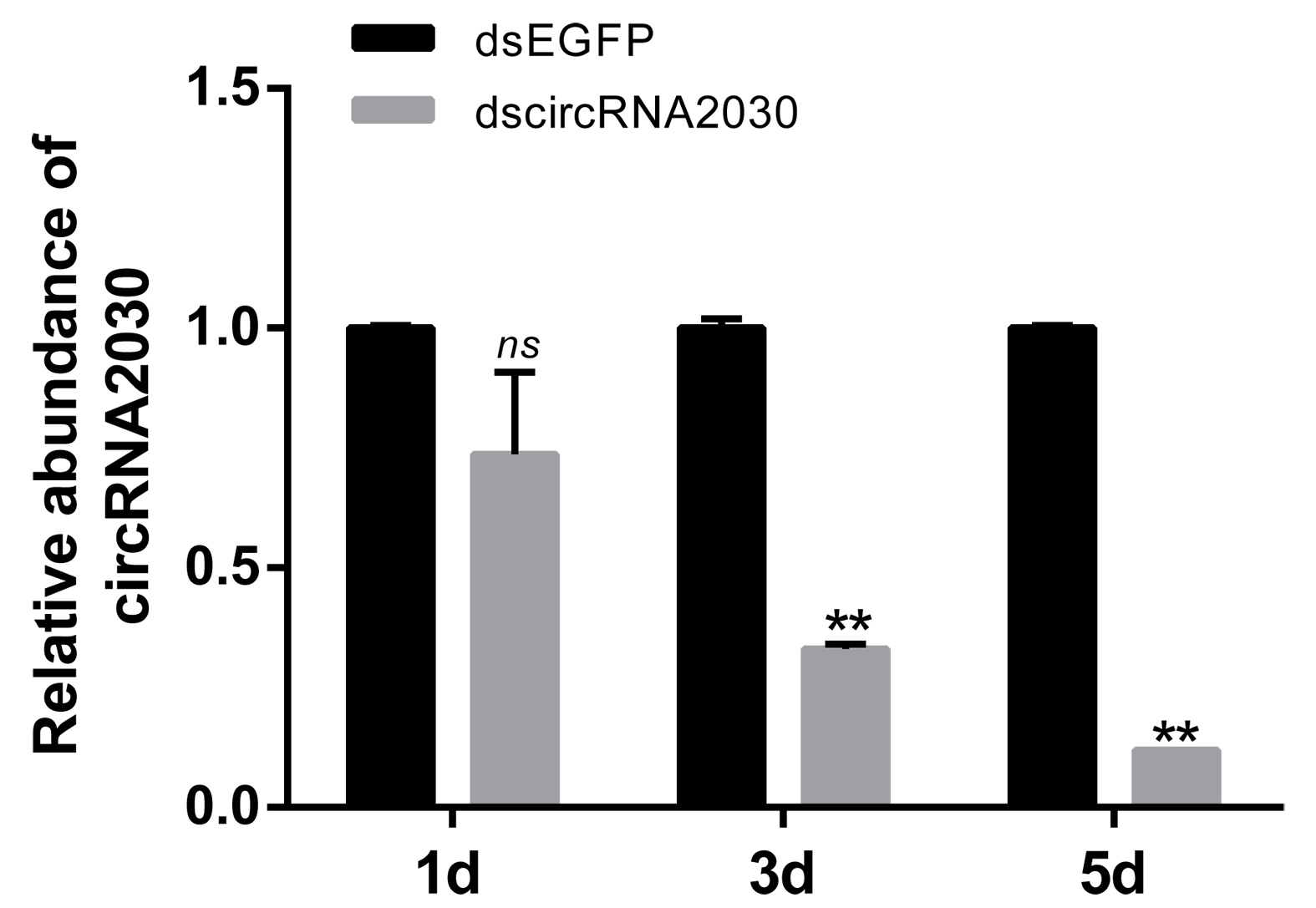

Supplement: Supplementary Figure 2 — Analysis of the expression level of circRNA2030 after dsRNA injection by RT-qPCR. The ns represents no significant difference, and the asterisks (∗∗) indicate significant differences at p < 0.01 levels. [file Image_2.TIF]
